# Solving genetic disease at the population scale

**DOI:** 10.1186/1471-2164-15-S2-O15

**Published:** 2014-04-02

**Authors:** Barry Merriman

**Affiliations:** 1 Lead System Architect, Advanced DNA Sequencing Technology, CSO, Enterprise Genomics Group, Life Technologies, Inc., USA; 2 Department of Human Genetics, UCLA, USA

## 

I will discuss the latest advances in genome sequencing technology, and the approach to the $1000 genome, with a focus on the status of the new Ion Torrent Proton Platform and how this can be applied in a systematic way to solve genetic diseases for entire populations. For this, I will present a process that identifies and removes all the bottlenecks in deploying genomic personalized medicine for entire countries. Examples will be provided based on specific national efforts already underway, in the USA and globally, and I will discuss the requirements that make countries or regions good candidates for this approach, and the opportunities for such countries to begin or accelerate the process.

